# Large-scale synthesis of graphene and other 2D materials towards industrialization

**DOI:** 10.1038/s41467-022-29182-y

**Published:** 2022-03-18

**Authors:** Soo Ho Choi, Seok Joon Yun, Yo Seob Won, Chang Seok Oh, Soo Min Kim, Ki Kang Kim, Young Hee Lee

**Affiliations:** 1 Center for Integrated Nanostructure Physics, Institute for Basic Science (IBS), Suwon, 16419 Republic of Korea; 2 Department of Energy Science, Sungkyunkwan University, Suwon, 16419 Republic of Korea; 3 Department of Chemistry, Sookmyung Women’s University, Seoul, 14072 Republic of Korea

**Keywords:** Electronic devices, Synthesis of graphene, Two-dimensional materials, Synthesis and processing

## Abstract

The industrial application of two-dimensional (2D) materials strongly depends on the large-scale manufacturing of high-quality 2D films and powders. Here, the authors analyze three state-of-the art mass production techniques, discussing the recent progress and remaining challenges for future improvements.

## Teaser

The effective application of graphene and other 2D materials is strongly dependent on the industrial-scale manufacturing of films and powders of appropriate morphology and quality. Here, we discuss three state-of-the-art mass production techniques, their limitations, and opportunities for future improvement.

**Fig. 1:**
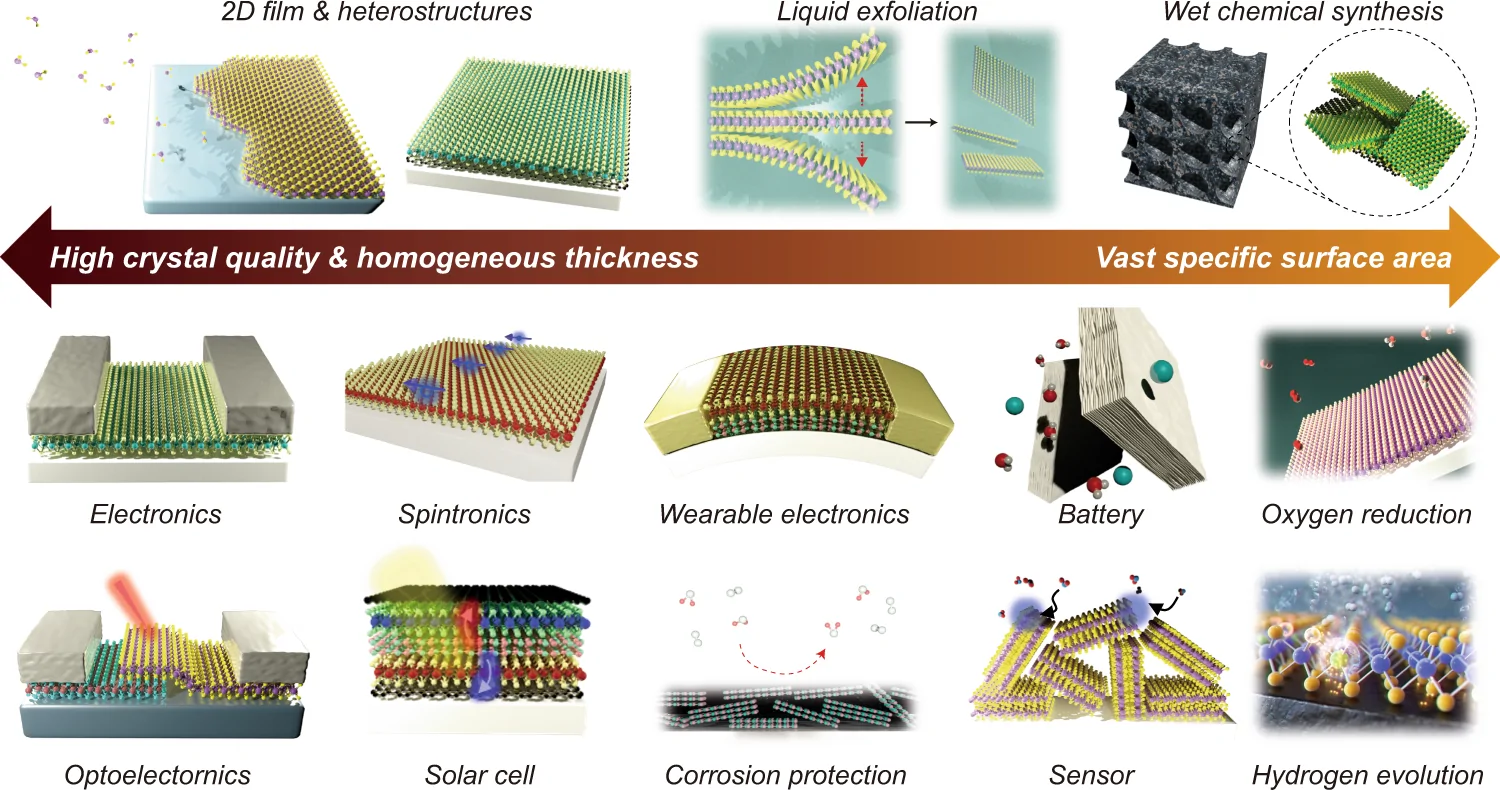
Specific target-oriented techniques for the mass production of 2D materials. 2D films and heterostructures require high crystal quality and homogeneous thickness for applications such as electronics and spintronics, whereas high-porosity powders with vast specific surface area can be used in contexts such as catalysts and energy storage.

Two-dimensional (2D) van der Waals (vdW) layered materials including graphene, transition metal dichalcogenides (TMDs), hexagonal boron nitride (hBN), and MXenes have attracted much attention in recent years. This is due to their distinctive physical and chemical properties, such as their quantum Hall effects and quantum valley Hall effects, indirect-to-direct bandgap transition, and strong spin-orbit coupling^1^, which have not been accessible with conventional 3D bulk materials. In addition, vertical vdW heterostructures constructed by layer-by-layer stacking enable interesting applications for atomically thin quantum wells, p-n junctions, Coulomb drag transistors, and twistronic devices^1–3^. However, applications based on such structures are limited by the fact that most vdW materials are currently only available with a lateral size of up to a few tens of micrometers. Techniques for the large-scale synthesis of 2D materials will therefore be required for industrialization. Moreover, since specific applications of these materials are strongly dependent on characteristics such as their morphology and quality, mass production techniques should also be developed that can accommodate such requirements (Fig. 1). In general, most applications rely on either films or powders of vdW materials. Films require high crystal quality, and can be used in the context of electronics, spintronics, optoelectronics, twistronics, or solar cells, whereas powders exhibit large surface areas and are employed in the construction of batteries, sensors, and catalysts. At present, only large-area graphene films and graphite oxide powders are currently available in the commercial market^4^. In this Comment, we briefly examine research trends in synthesis techniques and their associated challenges for the industrialization of 2D layered materials.

## Three representative synthesis techniques

There are currently three representative synthesis techniques available for the large-scale synthesis of 2D materials. The first is chemical vapor deposition (CVD); although a variety of thin-film deposition techniques have been investigated for growing large-area 2D films, including pulsed laser deposition (PLD)^5^, atomic layer deposition (ALD)^6^, and molecular beam epitaxy (MBE)^7^, CVD is most feasible for industrialization when one takes into account the uniformity and crystallinity of 2D films as well as requirements of high throughput, cost effectiveness, and scalability. The other two techniques being investigated for mass production are top-down liquid exfoliation of 2D materials and bottom-up wet chemical synthesis.

## CVD for growing large-scale 2D thin films

**Fig. 2:**
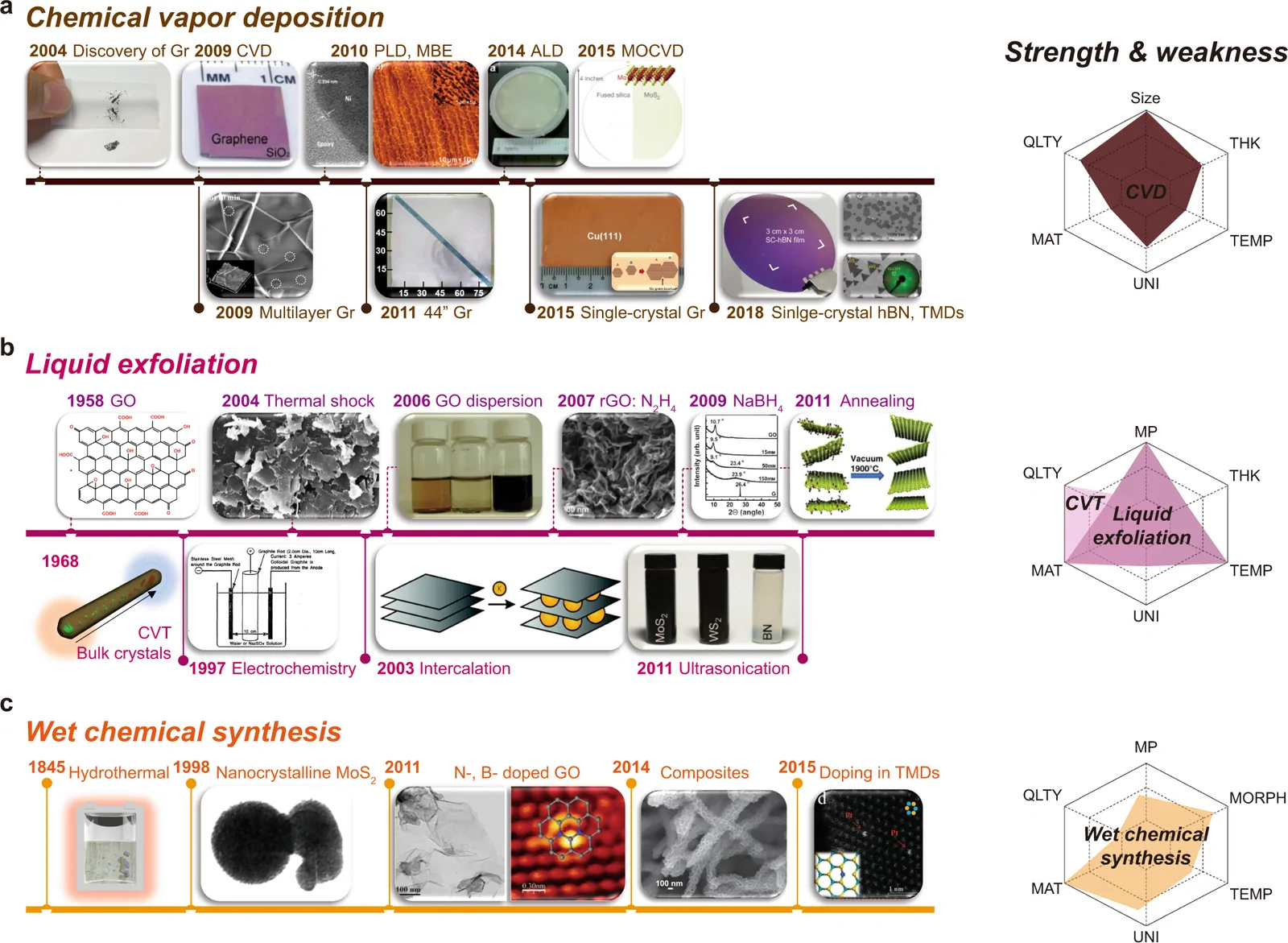
Historical landmarks in the development of three representative synthesis techniques. Lefthand panels show timelines of milestones for **a** chemical vapor deposition (CVD), **b** liquid exfoliation, and **c** wet chemical synthesis methods. The abbreviations correspond to: metal-organic CVD (MOCVD), graphene (Gr), graphite oxide (GO), reduced GO (rGO), and molybdenum disulfide (MoS_2_). Righthand panels show the corresponding strengths and weaknesses of these methods in terms of mass production (MP), thickness controllability (THK), temperature variation (TEMP), uniformity (UNI), material diversity (MAT), crystal quality (QLTY), morphology (MORPH). Panel a reprinted from refs. ^9,17^, American Association for the Advancement of Science, ref. ^15^, Nature, refs. ^7,10,12^, Wiley, ref. ^5^, American Institute of Physics, ref. ^6^, Royal Society of Chemistry, and ref. ^11^, World Scientific. Panel **b** reprinted from refs. ^26,27^, American Association for the Advancement of Science, refs. ^22,23^, Wiley, refs. ^19–21^, Elsevier, and ref. ^24^, Institute of Electrical and Electronic Engineers. Panel **c** reprinted from ref. ^28^, Elsevier, ref. ^30^, American Chemical Society, ref. ^31^, Elsevier, and ref. ^32^, Royal Society of Chemistry.

There are multiple examples of CVD synthesis of thin films at wafer scale (Fig. 2a). For example, wafer-scale polycrystalline monolayer and multilayer graphene films have been successfully synthesized by CVD on polycrystalline Cu and Ni foils since 2009^8–11^, and wafer-scale single-crystal monolayer graphene has been synthesized by using single-crystal substrates such as H–Ge (110) and Cu (111)^12,13^. Single-crystal multilayer graphene films have been also grown on Si–Cu alloys at wafer scale^14^. In 2012, centimeter-scale polycrystalline monolayers of hBN and TMDs were grown on polycrystalline Cu foils and SiO_2_/Si substrates, respectively^15,16^. And more recently, single-crystal hBN and TMD films were successfully synthesized on liquid Au, high-index single-crystal Cu surfaces, and atomic sawtooth Au surfaces^17,18^.

CVD produces relatively high-quality 2D films under atmospheric or low pressure, and the size of the film can easily be scaled up by increasing the chamber size. However, high temperature reactions (above 500 °C) are required, which could be a drawback for industrialization. The growth of a vast range of 2D materials, including graphene, hBN, and TMDs, is still limited by the absence of appropriate precursors. Perhaps the most important technical challenge presented by this method is the poor control over the number of synthesized layers, because the absence of dangling bonds on the surface of 2D vdW materials makes epitaxial growth difficult.

## Liquid exfoliation

Liquid exfoliation is a powerful process for the mass production of pristine 2D bulk materials by dispersing them into individual sheets. Bulk materials have been synthesized by chemical vapor transport (CVT) (Fig. 2b) since the late 1960s, but most 2D bulk materials are currently only available in small quantities. Nanodispersion into monolayers is often required to manifest the unique 2D nature, but the strong vdW energy of micron-scale materials hinders facile exfoliation. Thus, two additional steps should be considered for liquid exfoliation processes: (i) weakening the layer-layer interaction by expanding the interlayer distance, and (ii) physical agitation for dispersion^19,20^. In 1958, it was demonstrated that the interlayer distance can be increased from 3.4 to 7.0 Å by the oxidation of graphite, known as “graphite oxide”, and such an expansion of the interlayer distance made it possible to disperse the individual graphite oxide layers by sonication. Graphite oxide layers can subsequently be reduced to graphene nanosheets through chemical treatment with reducing agents and thermal annealing treatment^21–23^.

The lattice of graphene nanosheets is often severely damaged during oxidation and reduction processes. To prevent this, the interlayer distance can be increased by intercalating ions and molecules between layers. Electrochemistry enables effective intercalation of both cations and anions in an electrolyte solution by applying negative and positive bias, respectively^24^. Alkali metals, organic solvents, and surfactants with similar surface energies to those of the 2D materials can also be directly intercalated in liquid or vapor phase^25,26^. After intercalation, agitation methods such as sonication, homogenization, and microwave treatment can be employed to exfoliate materials into individual 2D layers^27^. Liquid exfoliation enables mass production of 2D nanosheets under atmospheric pressure at room temperature. However, this approach also leads to presumably unavoidable damage and non-uniform nanosheet thickness.

## Wet chemical synthesis

Hydrothermal and solvothermal syntheses are representative wet chemical synthesis methods, in which materials are respectively solubilized in aqueous solution and organic solvent under high vapor pressure at elevated temperatures (~300 °C). A variety of nanomaterials have been synthesized in this fashion since the first report of hydrothermal synthesis of microscopic quartz crystals in 1845 (Fig. 2c). The wet chemical synthesis of pure 2D materials such as graphene and MoS_2_ surged in the early 21st century^28,29^, and more recently, doped 2D materials, nanocomposites, and their heterostructures have been synthesized in this fashion by adding various precursors and dopants in solvent to enhance the material properties for specific applications^30–33^. For example, the hydrogen evolution reaction in graphene oxide was dramatically enhanced by introducing boron dopants^33^.

The strengths of wet chemical synthesis include the controllability of surface morphology, crystallite size, and dopants in 2D materials for catalyst, energy storage, and chemical/biological sensor applications. Reaction temperatures, precursors, and additives have been optimized for various types of 2D materials and their composites, enabling essentially unlimited mass production. The direct synthesis of 2D materials on a desired substrate is also possible, although such synthesis takes a relatively longer time—up to a few days. Growth temperature is often limited to below 300 °C due to the limited durability of equipment under harsh conditions including high pressure and exposure to corrosive chemicals. It is worth noting that bottom-up synthesis tends to yield low-quality 2D materials with defects, but it is still possible to employ these for catalytic applications.

## Perspectives and challenges toward industrialization

**Fig. 3:**
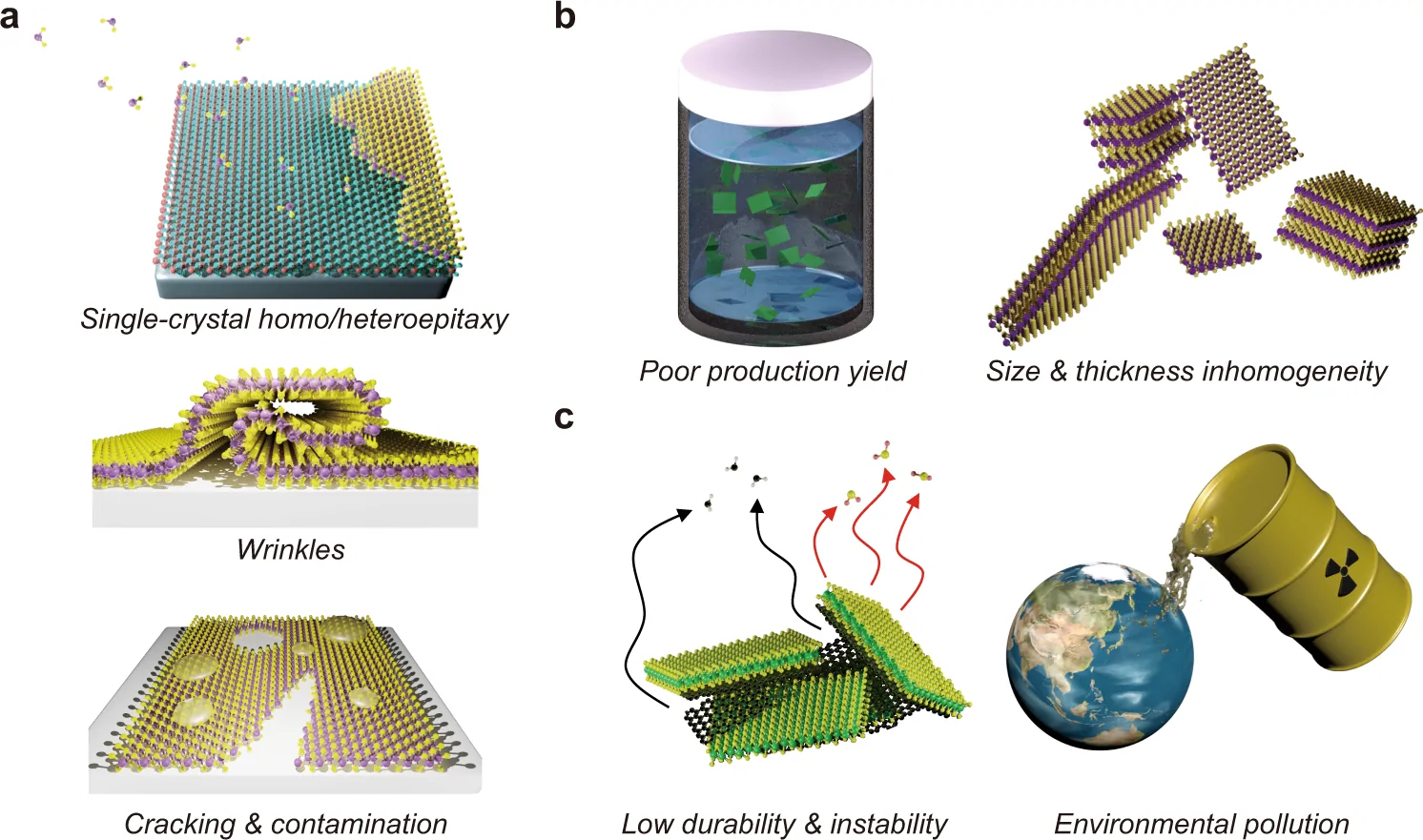
Current challenges in the mass production of 2D materials. **a** Single-crystal homo/heteroepitaxial growth, wrinkle formation by thermal expansion coefficient mismatch between 2D materials and growth substrates, and cracking/contamination during the transfer process are all issues presented by the CVD technique. **b** Inhomogeneous size and thickness of 2D nanosheets and poor production yield are problems associated with liquid exfoliation. **c** Low durability and instability of 2D materials by defects and environmental pollution remain challenges for wet chemical synthesis.

The abovementioned techniques enable mass production of 2D materials, but considerable further advances will be required for some specific applications. Single-crystal graphene films have been successfully synthesized at wafer scale with layer control, but the synthesis of other 2D materials such as hBN and TMDs are limited exclusively to single-crystal monolayer films. Thickness control of such materials is essential for tunneling barrier and high-performance electronics. The combination of tunable bandgap semiconductors, metals, and insulators in 2D systems can generate versatile heterostructures with remarkable physical properties. Several planar and vertical heterostructures have been generated to date, but these remain limited to micron scale^34,35^. More generally, the growth of various heterostructures at wafer scale is still challenging (Fig. 3a). Atomic sawtooth surfaces could be ideal as a growth platform for single-crystal 2D materials including graphene, hBN, TMDs, and their heterostructures, but surface facet control remains elusive. The formation of wrinkles in 2D films after high temperature growth is another important issue, originating from the thermal expansion coefficient mismatch between 2D materials and growth substrate. Recently, the growth of fold-free single-crystal graphene films at 750 °C has been reported^36^, but further investigation will be required to see if this method is applicable for other 2D materials, and lower-temperature growth methods should be established.

High temperature processes (above 400 °C) are not compatible with current Si technology, and 2D thin films grown by CVD at high temperature must therefore be transferred onto a target substrate. A conventional transfer process can give rise to serious problems such as folding and cracking of 2D films, ultimately degrading film homogeneity and device performance. Furthermore, the polymer contaminants that are commonly introduced as a supporting layer for the transfer process can give rise to unintentional doping and high contact resistance in heterostructure interfaces and devices. Therefore, methods for the direct growth of large-area 2D films by CVD or advanced roll-to-roll transfer technique would be highly desirable. For industrialization, the manufacturing process including scalable techniques (roll-to-roll, batch-to-batch, etc.), production capacity/cost, reproducibility, and large-area uniformity are further considered^37^.

Wet chemical processes including liquid exfoliation and wet chemical synthesis also face several challenges for the mass production of 2D materials. Liquid exfoliation employs pristine 2D bulk materials synthesized by CVT or flux methods for the mass production of 2D nanosheets. Those synthetic methods typically take at least one week, lowering the throughput of production, and companies need the capacity to provide these bulk materials at a larger scale. Additionally, the production yield of liquid exfoliation generally remains poor, and although some materials show relatively high yield, most 2D materials like hBN and telluride are not effectively exfoliated with current techniques. In addition, it is difficult to obtain 2D nanosheets of uniform size and thickness with this method (Fig. 3b). In order to remedy this, improved techniques for sorting the synthesized nanosheets in terms of size and thickness (e.g., density gradient ultracentrifugation) are needed.

Bottom-up chemical synthesis typically produces 2D materials with low crystal quality. The defect sites (i.e., edges) often serve as active sites for 2D catalyst, but also give rise to low durability and instability issues. Moreover, 2D materials generated by chemical synthesis are not uniformly distributed in terms of size and thickness, requiring special care during synthesis. In addition, the byproducts frequently generated during chemical synthesis can inhibit catalytic activity. To resolve these material quality and byproduct issues, post-treatments such as thermal annealing and purification have been suggested, but a simple process without post-treatment would greatly improve productivity. Another important issue is the environmental pollution caused by the large amount of hazardous chemical wastes used in synthesis (Fig. 3c), and the use of supercritical fluid regions could be considered as a shortcut to minimize chemical use^38^.

In addition, rapid and reliable non-destructive characterization tools are highly required to evaluate the wafer-scale 2D materials in terms of sample quality and uniformity. The current state-of-the-art terahertz image, phase-shift interferometry, and wide-field Raman imaging could be adopted to analyze the electrical and optical properties of 2D films with short acquisition time of a few seconds per mm^2^ and high spatial resolution of an order of micrometers^39–41^. It still requires a prolonged period to thoroughly inspect 12-inch wafer-scale sample, and therefore, the development of advanced characterization tools is further desired.

From a materials point of view, there is plenty of room for unexplored novel 2D materials and their vdW heterostructures. Since it is almost impossible to explore all such materials experimentally, artificial intelligence-based material design could prove useful for the industrialization and large-scale manufacture of such newly-developed 2D materials^42^.
